# Supplementary material for: Chromothripsis during telomere crisis is independent of NHEJ, and consistent with a replicative origin
Source: Genome Res. 2019 May;29(5):737–49. doi: 10.1101/gr.240705.118 (PMC6499312; doi:10.1101/gr.240705.118)
Supplement: Supplemental Material [file supp_gr.240705.118_Supplemental_file_1.zip › contigs/annotated_contigs/DB68/contig.2.DB68_length_831_mean_cov_13.7027677497.docx]

**DB68_length_831_mean_cov_13.7027677497**

```
ACAGTTCATGCTTTGATAATGCTAAAGTTGATAAGTAGATTAAGATGTGATAGAACAATCTGTAAATCTTAGTGCTTCCTAACTTTTGA
 >chr20:26204272-26204614 + E=6e-162
GAAAACATTTTTAGGCAAGATATGCTGATTTAAGGAAGAAAAGTTGCTAATGGATGAGGCATGTCCCTTAAAGACAAACACTATGAACA

CTGTGTACAGCCTTTATAATACAAAGATGCCAGGTTTTTTTTTAAGTCCTTGGAATCTCAGTTGGCAACTTTGAAATTCATGTGTAATT

CATGAGTTTTTTAAAAGAAATGTAATAAGTAACACAAGTGATTCTAGAGCTTGTCTTTAGGCTGAGGTAGATA|T|ACTTCTTTTTTCC
                                                                          >chr20:262053
TGTTTATGCCAATTATTTCTATTCCTCTTTACATATAGACAGAGAGATATGACTAAAATGAGTCATAGACAACGACATCTACTAAATTT
71-26205830 + E=4e-207
ACCTATTTTCATGTTGAATTCCACAACAGATTTCAAATACAACCAAAATCTCCAAATGAAATCTTAATTCTAAATTGAGTGCTTGTAGT

CTTCAAATATGTTCAAATAGAACATCAAAGCTCAATAAAAACAGTTATATCACTTGGGATGGTATTTAAAATACTTTTTCCTAAATAAA

TTCAGCTTCAGAGGATGAGAAAACCTTCATTAGGAGATGTATCTTAAAGGACAAATTAAGTTAAACAAATGTGAAAGAAATCATACTTT

ATTATGTTGAATAGTAAGATACTTTTGTTACTATTGTGAATTAATGCCATCCAAATATTGAGTGATGTAATTCAATTTAGTAAAAGAAA

|GATCGGAAGAGCGTCGTGTAGGGAAAGAGTGT
```
